# Antibiotic resistance and associated gene mutations of *Helicobacter pylori* in children: a three-year retrospective study in Shanghai, China

**DOI:** 10.3389/fcimb.2026.1839279

**Published:** 2026-06-04

**Authors:** Chunling Li, Yonghui Lu, Keyue Zhu, Leiyan He, Pan Fu, Saige Chen, Jie Qin, Ying Huang, Chuanqing Wang

**Affiliations:** 1 Lab of Microbiology, Department of Clinical Laboratory, Children’s Hospital of Fudan University, National Children’s Medical Center, Shanghai, China; 2 Department of Infectious Diseases, Taizhou Hospital of Zhejiang Province affiliated to Wenzhou Medical University, Shanghai, China; 3 Wenzhou Medical University, School of Laboratory Medicine and Life Science, Shanghai, China; 4 Nosocomial Infection Control Department, Children’s Hospital of Fudan University, National Children’s Medical Center, Shanghai, China; 5 Department of Gastroenterology, Children’s Hospital of Fudan University, National Children’s Medical Center, Shanghai, China

**Keywords:** *Helicobacter pylori*, children, antibiotic resistance, gene mutations, 16S *rRNA*, retrospective study

## Abstract

**Background:**

The changes in the antibiotic resistance patterns of *Helicobacter pylori (H*. *pylori)* are critical for guiding clinical eradication therapy. However, data on antibiotic resistance in *H. pylori* strains isolated from children in Shanghai have been unavailable for the past three years.

**Methods:**

676 *H. pylori* strains isolated from children and relevant clinical information was collected. Antibiotic susceptibility of metronidazole, clarithromycin, levofloxacin and amoxicillin was tested by E-test, resistance gene mutations were detected via PCR and sequencing.

**Results:**

Among 676 *H. pylori* isolates, total resistance rates to metronidazole, clarithromycin, levofloxacin and amoxicillin were 61.5%, 39.1%, 18.2% and 0.3%, respectively. Resistance rates to metronidazole, clarithromycin increased significantly from 2023 to 2025, with increased dual and hetero-resistance. Higher rates of resistance to metronidazole and levofloxacin were observed in the 7–12 year-old group than other two age groups. A2143G in 23S *rRNA* (98.7%) and N87K in *gyrA* (61.0%) were the main mutations for clarithromycin and levofloxacin resistance.

**Conclusion:**

Over the past three years, a substantial increase in antibiotic resistance toward metronidazole and clarithromycin is observed in *H. pylori* isolated from children in Shanghai, China.

## 1 Introduction

*Helicobacter pylori* (*H*. *pylori*) infection in children is closely associated with many health problems such as: chronic gastritis, peptic ulcers, and stunted growth (Baj et al., 2021; Tshibangu-Kabamba and Yamaoka, 2021; Salahi-Niri et al., 2024). Metronidazole (MTZ), clarithromycin (CLA), levofloxacin (LEV) and amoxicillin (AML) are commonly used core drugs for *H. pylori* eradication therapy. However, there has been a notable increase in treatment failure rates in recent years, attributable to the ongoing rise in antimicrobial resistance (Li et al., 2024; Yu et al., 2024). This poses a significant clinical challenge, underscoring the necessity for a more comprehensive understanding of antibiotic resistance in *H. pylori*.

Despite the clinical importance of understanding resistance trends to guide empirical therapy, comprehensive surveillance data on antibiotic resistance profiles among pediatric *H. pylori* isolates in Shanghai remain scarce. Moreover, molecular information related to resistance−associated gene mutations in these pediatric strains is even more limited, which hinders the development of rapid molecular diagnostic methods and optimized treatment strategies for children. Therefore, updated investigations into the phenotypic antibiotic susceptibility and underlying genotypic mechanisms of *H. pylori* in the pediatric population of Shanghai are urgently needed to improve clinical management and combat the growing problem of drug resistance.

Researches had indicated that point mutations of the *23S rRNA* gene (particularly A2143G, A2142G and A2142C mutations) and *gyrA* are associated with *H. pylori* resistance to CLA and LEV. Furthermore, it has been demonstrated that mutations in the *rdxA, frxA*, and *frxB* genes have a pivotal function in MTZ resistance (Marques et al., 2019; Vieni et al., 2025; Martínez-Martínez et al., 2026). In addition, mutations in the *pbp1A* gene, which codes for penicillin-binding proteins (PBPs), have been reported to play a pivotal role in AML resistance, as well as the outcomes of eradication therapy (Matta et al., 2023; Vieni et al., 2025).

The objective of this study was to systematically characterize the antibiotic resistance phenotypes and corresponding resistance gene mutations among *H. pylori* isolates recovered from pediatric patients in Shanghai, China, with a specific focus on CLA, MTZ, LEV, and AML resistance genotypes. Through genotypic-phenotypic correlation analyses and regional mutation profiling, this work will help to establish a molecular epidemiological framework to guide evidence-based treatment strategies and optimize infection control measures, addressing the current knowledge gap in pediatric antimicrobial stewardship.

## 2 Methods

### 2.1 Study design

A total of 676 *H. pylori* isolates in this study were obtained from children with upper gastrointestinal diseases from January 2023 to December 2025 in Children’s Hospital of Fudan University.

### 2.2 *H. pylori* culture and antibiotic susceptibility tests

The gastric mucosa biopsy sample should be immediately placed into a transport tube containing 5ml Brucella broth (Oxoid, Dardilly, France), and then sent to the laboratory as soon as possible with a 4°C transfer box.

Gastric mucosa biopsy samples were homogenized in 0.5 ml Brucella broth (Oxoid, Dardilly, France) and inoculated on supplemented Columbia agar (Oxoid). These plates were inoculated at 37°C for 3 to 7 days under microaerophilic conditions (5% O_2_, 10% CO_2_, and 85% N_2_). Characteristic small transparent or translucent colonies of *H. pylori* were identified through modified Gram staining (Baso Biotec. Zhuhai), rapid urease reactions, and by conducting catalase, oxidase, and nitrate reduction tests (Mégraud and Lehours, 2007).

The antibiotic susceptibility of all *H. pylori* isolates to metronidazole (MTZ), clarithromycin (CLA), amoxicillin (AML), and levofloxacin (LEV) was determined using the E-test method (Taikang Bio, China), following the Clinical and Laboratory Standards Institute (CLSI, Wayne, PA, USA) reference guidelines. Strains were recovered after two subculture passages and suspended in physiological saline to achieve a turbidity equivalent to the 2.0 McFarland standard. Each standardized suspension was uniformly inoculated onto Mueller-Hinton agar plates (Oxoid) supplemented with 10% (v/v) defibrinated sheep blood. E-test strips containing MTZ, CLA, AML, and LEV were applied to the inoculated plates, which were subsequently incubated at 37 °C for 72 hours under microaerophilic conditions (5% O_2_, 10% CO_2_, 85% N_2_) (Mégraud and Lehours, 2007). Drug resistance breakpoints were defined as follows: MIC > 0.125 μg/mL for AML, MIC > 1 μg/mL for LEV, MIC > 0.5 μg/mL for CLA, and MIC > 8 μg/mL for MTZ.

### 2.3 Molecular detection on antibiotic resistant *H. pylori* strains

All primers sequences used in this study were derived from published research results (**Table 1**) and synthesized at Maipu Biotech Co., Ltd. (Shanghai, China) (Wang et al., 2020; Tran et al., 2022; Li et al., 2024).

**Table 1:** Primer sequences for molecular detection of *H. pylori.*.

Gene	Primers’ sequence (5′-3′)	Amplicon length (bp)	Reference
16S *rRNA*	F: TAAGAGATCAGCCTATGTCC	534	(Li et al., 2024)
	R: TCCCACGCTTTAAGCGCAAT		
23S *rRNA*	F: CCACAGCGATGTGGTCTCAG	425	(Wang et al., 2020)
	R: CTCCATAAGAGCCAAAGCCC		
*gyrA*	F: AGCTTATTCCATGAGCGTGA	582	(Wang et al., 2020)
	R: TCAGGCCCTTTGACAAATTC		
*pbp1A*	F: CGATAGATTTGGATTACCAACGC	1035	(Tran et al., 2022)
	R: ACGATTTCTTTACGCAAGCC		

Genomic DNA was isolated and purified from clinical isolates of *H. pylori* using a commercially available kit (Tiangen Biotech Co., Ltd., Beijing, China), strictly following the manufacturer’s protocol. The purified DNA served as a template for PCR amplification, enabling the systematic detection of mutation in *rdxA, gyrA, pbp1A* and *23S rRNA*. The PCR products were sequenced by Maipu Biotech Co., Ltd. Each sequence was then compared with the published sequence of the *H. pylori* strain 26695 (GenBank accession number AE000511.1) using the MEGA11 software.

### 2.4 Statistical analysis

Data were analyzed using SPSS software (version 26.0; SPSS Inc., Chicago, IL, USA). Differences in Proportion were evaluated using the Chi-square test. A *P*-value < 0.05 was considered statistically significant.

## 3 Results

### 3.1 Antibiotic resistance patterns of *H. pylori* isolates

Among 676 *H. pylori* isolates, total resistance rates to MTZ, CLA, LEV and AML were 61.5% (416/676), 39.1% (264/676), 18.2% (123/676) and 0.3% (2/676), respectively. 259 (38.3%) isolates were monoresistance to MTZ, CLA and LEV, among them, the most frequent for MTZ (29.3%, 198/676), followed by CLA (7.4%, 50/676) and LEV (1.6%, 11/676). Dual resistance (26.8%, 181/676) was most common for MTZ+CLA (18.6%), followed by CLA+LEV (4.1%) and MTZ+LEV (4.9%). Hetero-resistance was detected in 9.3% (61/676) of isolates for MTZ+CLA+LEV and 0.3% (2/676) for MTZ+CLA+LEV+AML (**Table 2**). Among the 676 *H. pylori* strains, only 170 (25.1%) were sensitive to all antibiotics.

**Table 2:** Antibiotic resistance patterns of *H. pylori* isolates.

Resistance patterns	Number (%)
Total resistance	
MTZ	416 (61.5)
CLA	264 (39.1)
LEV	123 (18.2)
AML	2 (0.3)
Sensitive	170 (25.1)
Monoresistance	
MTZ	198 (29.3)
CLA	50 (7.4)
LEV	11 (1.6)
Dual resistance	
MTZ+CLA	126 (18.6)
MTZ+LEV	28 (4.1)
CLA+LEV	33 (4.9)
Hetero-resistance	
MTZ+CLA+LEV	61 (9.0)
MTZ+CLA+AML	2 (0.3)

### 3.2 Demographic characteristics of *H. pylori* isolates

Overall, 396 (58.6%) strains isolated from male and 280 (41.4%) from female. 98 (14.5%) strains were isolated from 1–6 years-old patients, 401(59.3%), 177 (26.2%) strains were isolated from 7–12 years-old patients and 13–17 years-old patients, respectively. Gender and age were analyzed as potential factors that may influence antibiotic resistance. We found that the resistance rate of *H. pylori* isolated from 7–12 year-old group was higher than other two age groups, especially to MTZ (*P* < 0.001) and LEV (*P* = 0.036).

Besides, there was no significant difference in the resistance rates to MTZ, CLA, LEV, AML between male and female (**Table 3**).

**Table 3:** Demographic characteristics of antimicrobial resistance of *H. pylori* isolates.

Antibiotic	Gender		*P*	Age, years			*P*
	Male (n=396)	Female (n=280)		1-6 (n=98)	7-12 (n=401)	13-17 (n=177)	
MTZ	244 (36.1)	172 (25.4)	0.961	51 (7.5)	241 (35.7)	124 (18.3)	<0.001
CLA	149 (22.0)	115 (17.0)	0.366	42 (6.2)	150 (22.2)	72 (10.7)	0.12
LEV	64 (9.5)	59 (8.7)	0.103	30 (4.4)	62 (9.2)	31 (4.6)	0.036
AML	1 (0.1)	1 (0.1)	\	0 (0)	1 (0.1)	1 (0.1)	\

### 3.3 Variation of antibiotic resistance of *H. pylori* isolates during 2023-2025

**Figure 1:**
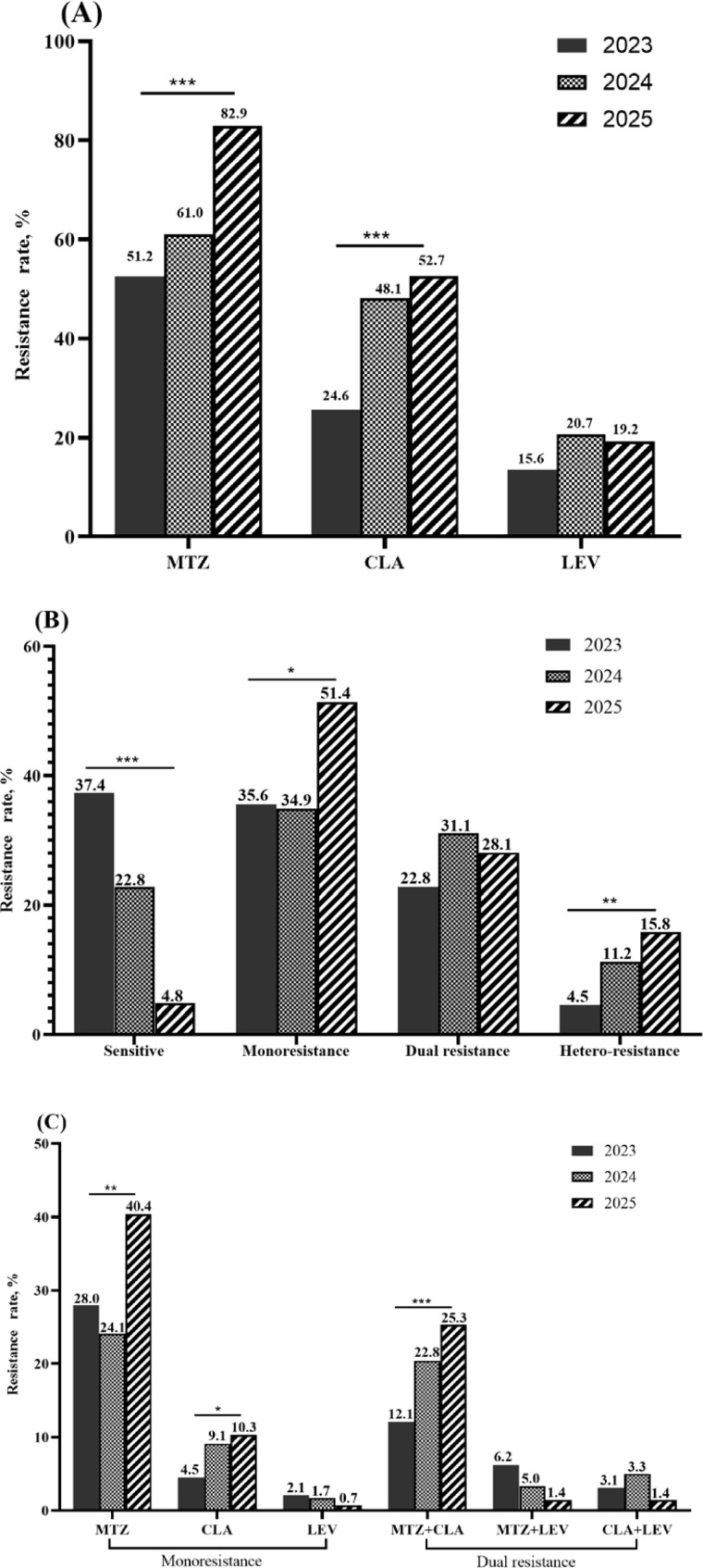
**(A)** Total antibiotic resistance rates to MTZ, CLA and LEV during 2023-2025. **(B)** Overall multiple antibiotic resistance rates of different resistance patterns during 2023-2025. **(C)** Antibiotic resistance rates of different resistance patterns detailed during 2023-2025. *P < 0.05, **P < 0.01, ***P < 0.001.

Our data showed that the resistance rate of 676 *H. pylori* isolates increased significantly between 2023 and 2025. The overall antibiotics resistance rate to MTZ, CLA were increased from 51.2% in 2023 to 82.9% in 2025 (*P* < 0.001), from 24.6% in 2023 to 52.7% in 2025 (*P* < 0.001), respectively (**Figure 1A**).

A significantly (*p*<0.001) noticeable increase from 4.5% to 15.8% in MTZ+CLA+LEV based hereto-resistance was observed from 2023 to 2025. (*P* < 0.001) (**Figure 1B**). MTZ+CLA+LEV was the only hetero-resistance pattern.

The dual resistance rates increase from 35.6% in 2023 to 51.4% in 2025 with *p* = 0.036 (**Figure 1B**). Among them, the MTZ+CLA pattern showed the most significant increase in drug resistance, from 12.1% in 2023 to 25.3% in 2025 with *P* = 0.01 (**Figure 1C**). For the monoresistance *H. pylori* isolates, antibiotics resistance rate to MTZ, CLA were increased from 28.0% in 2023 to 40.4% in 2025(*P* = 0.002), from 4.5% in 2023 to 10.3% in 2025(*P* = 0.041)(**Figure 1C**).

It is worth noting that the rate of sensitive strains decreased from 37.4% to 4.8%, during 2023 and 2025(*P* < 0.001). (**Figure 1A**).

### 3.4 Genotype profiles of antibiotic *H. pylori* strains

We randomly selected 150 (50 strains per year from 2023 to 2025) *H. pylori* strains resistant to CLA, and then analyzed CLA-associated resistance mutation sites by 23S *rRNA* sequencing. A2143G point mutation was observed in 148 (98.7%) cases among the 150 strains, which is the most predominant mutation type of CLA-related drug resistance. Only 2 (1.3%) cases were found in A2142G point mutation (**Table 4**).

**Table 4:** Antibiotic resistance associated mutations of *H. pylori* strains.

Mutation type	Number (%)
CLA(*23S rRNA*) n=150	
A2142G	2 (1.3)
A2143G	148 (98.7)
LEV(*gyrA*) n=123	
N87I	9 (7.3)
N87K	75 (61.0)
D91N	18 (14.6)
D91G	2 (1.6)
D92Y	16 (13.0)
None	3 (2.4)
AML(*PBP1A*) n=2	
P366L	2 (100)
A369T	0 (0)

Among 123 LEV associated resistance *H. pylori* stains, 120 (97.6%) cases revealed *gryA* amino acid sequences changes. The N87K point mutation was the most prevalent, occurring at 61.0% of the de mutation sites. The D91N and D92Y mutations followed, with frequencies of 14.6% and 13.0%, respectively (**Table 4**). For 2 cases of AML resistance *H. pylori* stains, we measured the *PBP1A* sequence and found that both cases were P366L amino acid locus mutations (**Table 4**).

## 4 Discussion

This study systematically characterized the antibiotic resistance phenotypes and corresponding resistance-associated gene mutation profiles of *H. pylori* isolates from pediatric patients, with a focus on four clinically critical antibiotics (metronidazole, clarithromycin, levofloxacin and amoxicillin), and the molecular resistance profiles of *H. pylori* in pediatric patients from Shanghai between 2023 and 2025.

Metronidazole is the classic antibiotic for the eradication of *H. pylori*. However the resistance rates of metronidazole are much higher in developing countries. However, in China, metronidazole resistance is high, typically ranging from 40% to 80%, far exceeding the 20-30% typical in Europe and the United States (Shu et al., 2018; Geng et al., 2022; Jiang et al., 2022; Tang et al., 2022; Salahi-Niri et al., 2024; Yu et al., 2024; Menbari et al., 2025; Xue et al., 2025; Zhang et al., 2025).The situation is further exacerbated by the high prevalence of metronidazole resistance among *H. pylori* isolates from children in Shanghai, which reached 61.5% over the past three years. This rate increased from 51.2% in 2023 to 82.9% in 2025, higher than earlier reported in 2022 (Li et al., 2024).According to the Maastricht/Florence consensus, a metronidazole resistance rate exceeding 40% substantially diminishes the efficacy of regimens containing this drug (Jukic et al., 2020). Consequently, metronidazole-based regimens should be used with caution or replaced with alternative agents. In clinical practice, when local resistance rates exceed 50%, metronidazole is generally not recommended as a first-line therapy. However, the resistance mechanism of metronidazole is complex, and although the *in vitro* resistance rate is high, clinical studies have shown that it still has a good eradication effect on HP when combined with bismuth (Kori et al., 2020).

Inactivation of the *rdxA/frxA* gene leading to loss of nitro-reductase function is the main mechanism of MTZ resistance (Tshibangu-Kabamba and Yamaoka, 2021). However, metronidazole resistance correlates poorly with specific genetic alterations, so we excluded metronidazole resistance genes from our analysis.

Clarithromycin remains the core antibiotic for *H. pylori* eradication, yet its widespread use has driven a significant global increase in resistance rates. Data for the year 2025 shows that the primary resistance rate has reached 23.4% in Italy, 13.8% in Seoul, South Korea, and the resistance rate in Beijing, China, has climbed from 10.0% in 1999 to 23.3% in 2024 (Salahi-Niri et al., 2024; Yu et al., 2024; Menbari et al., 2025; Zhang et al., 2025). Notably, resistance rates in pediatric populations (e.g., 29.0% in Japanese children) were generally higher than those in adults, suggesting that early exposure to medications may exacerbate the risk of resistance (Okimoto et al., 2024).In this study, the resistance rate to clarithromycin was 39.1% overall, having risen significantly from 24.6% in 2023 to 52.7% in 2025. which is shows an increasing trend as compared to earlier reports in 2022 (Li et al., 2024).

In addition, the rate of metronidazole + clarithromycin dual resistance has also increased significantly, from 12.1% in 2023 to 25.3% in 2025. According to the Maastricht/Florence consensus, the eradication rate of the non-bismuth quadruple regimen (PPI+ amoxicillin + clarithromycin + metronidazole) declines when dual resistance to metronidazole and clarithromycin exceeds 15%. Consequently, substituting the bismuth quadruple regimen (PPI+ bismuth+ two antibacterial drugs) is recommended to improve efficacy.

Site-specific 23S rRNA mutations (e.g., A2143G, A2142G, A2142C) in drug-resistant strains result in altered ribosome conformation and decreased drug affinity, these mutations are the main causes of resistance to clarithromycin (Matta et al., 2023; Vieni et al., 2025).Among them, the A2143G mutation was the most common, with an eradication rate of only 48%, which was significantly lower than that of other mutation types (e.g.A2142G/A2142C has an eradication rate of 93%). In this study, A2143G point mutation was observed in 148 (98.7%) cases among the 150 strains, consistent with the reported data (Rimbara et al., 2008; Gareayaghi and Kocazeybek, 2022; Tran et al., 2022). What’s more, we should pay much attention to a novel mutation site T2717C in Italy, which is located in the 23S rRNA VI region and causes low-level resistance (MIC = 1 μg/mL) by affecting the structure of the drug binding pocket (Rimbara et al., 2008).

Levofloxacin is a type of fluoroquinolone antibiotic drug. Based on Chinese expert consensus, regimens containing levofloxacin are not recommended as first-line therapy. In the present study, the observed resistance rate was 18.2%. It was reported that the resistance rate of *H. pylori* isolates to levofloxacin has reached 20–60% in China (Shu et al., 2018; Geng et al., 2022; Jiang et al., 2022; Geng et al., 2023; Xue et al., 2025; Huang et al., 2026). The resistance rate of *H. pylori* to levofloxacin in children is lower than that in adults, which may be due to the control of fluoroquinolone use in children.

Levofloxacin resistance in *H. pylori* arises primarily from mutations in the *gyrA* and *gyrB* genes, which encode subunits of DNA gyrase. These genetic alterations result in conformational changes that reduce the drug’s binding affinity for its target enzyme, thereby compromising its antimicrobial efficacy. In this study, *gyrA* was sequenced in 123 levofloxacin resistance *H. pylori* stains. The *gryA* amino acid sequence alterations were identified in 120 (97.6%) cases, and mutation at N87K (61.0%)) was predominantly in levofloxacin-resistant strains, similar to the reported data (Miftahussurur et al., 2016; Tang et al., 2022; Xue et al., 2025).

Global data show that the resistance rate of *H. pylori* to amoxicillin has increased from about 10% in 2010 to 20-30% in 2023, with higher rates in parts of Asia (e.g., China, India) (Salahi-Niri et al., 2024; Yu et al., 2024; Schulz et al., 2025). A study in Vietnam reported amoxicillin resistance at 25.7% (Tran et al., 2022).Only 0.3% (2/676) *H. pylori* strains was resistant to amoxicillin in this study, though the resistance rate of *H. pylori* to amoxicillin in Chinese children at a low level, vigilance remains necessary to ensure the rational use of antibiotics.

Over last three years, the rate of dual resistance toward metronidazole and clarithromycin has been doubled i.e., a significant increase in resistance from 12.1% in 2023 to 25.3% in 2025. Our study will provide up-to-date and strongly support for *H. pylori* eradication therapy in children. The findings of this study not only provide novel insights into the molecular epidemiology of *H. pylori* resistance in the local pediatric population but also offer actionable guidance for optimizing clinical treatment strategies and strengthening antibiotic stewardship in pediatric practice. Due to the severe problem of metronidazole and clarithromycin resistance, we need to strengthen the regulation of antibiotic use in children and reduce non-essential macrolide prescriptions. And individualized treatment based on genetic testing for drug resistance should be given more attention and promoted. In addition, a regional antimicrobial resistance surveillance network is urgently needed to dynamically monitor the evolving trends in drug resistance and inform evidence-based treatment strategies.

A particular limitation of this study is that it did not explore the effects of social and family factors on *H. pylori* infection in children, nor did it investigate the prognosis of patients after treatment.

## Data Availability

The data supporting the conclusions of this article will be made available by the authors, without undue reservation.
